# Antidepressants and suicide risk in children and adolescents

**DOI:** 10.1192/j.eurpsy.2024.1112

**Published:** 2024-08-27

**Authors:** S. Riam, N. Baabouchi, H. kisra, F. laajili

**Affiliations:** Arrazi University Psychiatric Hospital, Rabat Faculty of Medicine and Pharmacy Mohammed V University, RABAT, Morocco

## Abstract

**Introduction:**

In recent years, the prescription of antidepressants for children has faced significant scrutiny due to studies suggesting an elevated risk of suicide among those treated with these medications. The primary objective of this study is to examine the causal connection between antidepressant use and suicidal behavior in children and adolescents.

**Objectives:**

In this article, we will examine the current research on this topic and discuss the current status of practical guidelines and recommendations for prescribing antidepressants to children and adolescents.

**Methods:**

We conducted a literature review using the Google Scholar database, employing keywords such as antidepressants, suicide, children, and adolescents.

**Results:**

The literature yielded conflicting data. While it has been established that SSRIs moderately elevate the risk of suicide ideation and attempts, with venlafaxine, paroxetine, and sertraline showing a higher risk compared to other SSRIs like fluoxetine and citalopram, several studies indicate that their use is linked to a noteworthy reduction in suicide rates among children and adolescents.

**Conclusions:**

The existence of a definitive causal relationship between antidepressants and suicidality in children and adolescents is currently uncertain, and the underlying mechanisms remain inadequately understood.

**Disclosure of Interest:**

None Declared

